# Simulation of solute transport through heterogeneous networks: analysis using the method of moments and the statistics of local transport characteristics

**DOI:** 10.1038/s41598-018-22224-w

**Published:** 2018-02-28

**Authors:** Min Li, Tao Qi, Yves Bernabé, Jinzhou Zhao, Ying Wang, Dong Wang, Zheming Wang

**Affiliations:** 1 State Key Laboratory of Oil and Gas Reservoir Geology and Exploitation, Southwest Petroleum University, Chengdu, China; 2 Earth, Atmospheric and Planetary Sciences Department, Massachusetts Institute of Technology, Cambridge, Massachusetts, USA; 3 Pacific Northwest National Laboratory, 902 Battelle Boulevard, P. O. Box 999, MSIN K8-96, Richland, Washington, 99352 United States

## Abstract

We used a time domain random walk approach to simulate passive solute transport in networks. In individual pores, solute transport was modeled as a combination of Poiseuille flow and Taylor dispersion. The solute plume data were interpreted via the method of moments. Analysis of the first and second moments showed that the longitudinal dispersivity increased with increasing coefficient of variation of the pore radii *CV* and decreasing pore coordination number *Z*. The third moment was negative and its magnitude grew linearly with time, meaning that the simulated dispersion was intrinsically non-Fickian. The statistics of the Eulerian mean fluid velocities ${\hat{{\boldsymbol{u}}}}_{{\boldsymbol{i}}}$, the Taylor dispersion coefficients ${\hat{{\boldsymbol{D}}}}_{{\boldsymbol{i}}}$ and the transit times ${\hat{{\boldsymbol{\tau }}}}_{{\boldsymbol{i}}}$ were very complex and strongly affected by *CV* and *Z*. In particular, the probability of occurrence of negative velocities grew with increasing *CV* and decreasing *Z*. Hence, backward and forward transit times had to be distinguished. The high-τ branch of the transit-times probability curves had a power law form associated to non-Fickian behavior. However, the exponent was insensitive to pore connectivity, although variations of *Z* affected the third moment growth. Thus, we conclude that both the low- and high-τ branches played a role in generating the observed non-Fickian behavior.

## Introduction

Solute transport is a subject of great importance in fundamental physics as well as in applied disciplines such as hydrogeology (e.g., mixing of fresh and salt water in coastal aquifers), chemical engineering (e.g., use of reactors packed with granular aggregates) and petroleum engineering (e.g., secondary recovery techniques using miscible displacements). Although the continuum approach and the advection-dispersion equation^1,2^ (ADE) are still widely used today, a number of experimental and field studies exposed substantial deviations with respect to the ADE analysis, i.e., non-Fickian behavior^3–12^. Non-Fickian behavior can take different forms^11^. For example, data recorded during tracer experiments often indicate that the dispersion tensor needed for ADE modeling varies with time or, equivalently, with traveled distance. This type of behavior is expected during the transient (“pre-asymptotic”) regime preceding an eventual long-term Fickian regime. Another frequent indicator of non-Fickian behavior is asymmetry of the solute plume.

As a consequence, alternative modeling frameworks have been investigated. Probabilistic models inspired by the Fokker-Planck treatment of Brownian motion^13–16^ as well as models based on the critical path analysis of percolation theory^17–20^ were proposed. In this paper, we will focus on the probabilistic approach. Advection-dispersion is viewed as a stochastic process characterized by the conditional probability *P*(***x***, ***x***′, *t*, *t*′) of a solute particle transition from point ***x***′ at time *t*′ to ***x*** at *t*. The probability *P* is generally assumed stationary in time and space and can thus be written *P*(***s***, τ), with ***s*** = ***x*** − ***x***′ and τ = *t* − *t*′. This approach is particularly well suited to interpret the results of random walk/particle tracking simulations^21–28^. Random walk/particle tracking simulations have often been implemented in idealized representations of porous media such as networks of cylindrical pores^29–35^. The results of such network simulations can be conveniently analyzed using the method of moments. The second central longitudinal moment of the solute plume quantifies macroscopic dispersion and its linear growth with time is an operational indicator of the asymptotic regime. The third central moment provides a measure of the asymmetry of the solute plume.

Here, we report the results of time domain random walk simulations performed using a network simulation technique based on that of *Bernabé et al*.^35^ (this approach is poorly adapted to the study of fracture sets, which are thus not considered here despite their importance in field applications). The simulations were set in three-dimensional networks of cylindrical pores with randomly distributed radii. The rules governing the motion of solute particles were selected considering that advection and molecular diffusion are two transport mechanisms invariably present in experimental or field applications. The importance of molecular diffusion has often been questioned^1,8,11,17^. However, its interaction with a non-uniform fluid velocity field leads to a great enhancement of local spreading known as Taylor dispersion^36^. Accordingly, we assumed that solute transport in individual pores obeyed Taylor dispersion. Other mechanisms, such as those arising from, e.g., chemical interactions, may actually occur in real situations^11,19,20^, but were not included here for the sake of simplicity.

Thus, the simulations involved a cascade of four sets of random variables. The primary random variables are the pore radii, ${\hat{r}}_{i}$, followed in order of dependence by the Eulerian mean fluid velocities of individual pores, ${\hat{u}}_{i}$, the coefficients of Taylor dispersion, ${\hat{D}}_{i}$, and the transit times, ${\hat{\tau }}_{i}$. The pore radii ${\hat{r}}_{i}$ are assumed independent and spatially uncorrelated. They obey stationary and ergodic probability distributions. The pore fluid velocities are deterministically linked to the pore radii through the flow equations, i.e., the velocity realizations, *u*_*i*_, can be calculated from the radius realizations, *r*_*i*_, by application of mass conservation and Poiseuille equation. Notice that, owing to mass conservation^27^, realizations of ${\hat{u}}_{i}$ must display some amount of spatial correlation depending on the width of the pore radii distribution and the network connectivity (the pore flow rates ${\hat{q}}_{i}=\pi {\hat{r}}_{i}^{2}{\hat{u}}_{i}$ are the random variables most directly controlled by mass conservation). The coefficients of Taylor dispersion are also deterministically related to ${\hat{r}}_{i}$ and ${\hat{u}}_{i}$ by ${\hat{D}}_{i}={D}_{m}+\frac{{\hat{u}}_{i}^{2}{\hat{r}}_{i}^{2}}{C{D}_{m}}$, where *D*_*m*_ is the solute molecular diffusion coefficient and *C* a geometric constant, the value of which depends on the shape of the cross-section^36,37^ (*C* = 48 for a circular cross-section). Finally, the transit times ${\hat{\tau }}_{i}$ are stochastically generated from the preceding three variables, ${\hat{r}}_{i}$, ${\hat{u}}_{i}$ and ${\hat{D}}_{i}$, i.e., the realizations, τ_i_, are randomly produced according to the local cumulative distribution functions corresponding to Taylor dispersion^35^:

1 $$CDF({\tau }_{i})=\frac{1}{2}[{\rm{erfc}}(\frac{l-{u}_{i}{\tau }_{i}}{2\sqrt{{D}_{i}{\tau }_{i}}})+\exp (\frac{l{u}_{i}}{{D}_{i}}){\rm{erfc}}(\frac{l+{u}_{i}{\tau }_{i}}{2\sqrt{{D}_{i}{\tau }_{i}}})]$$

where *l* denotes the length of the pores. The first three variables, ${\hat{r}}_{i}$, ${\hat{u}}_{i}$ and ${\hat{D}}_{i}$, characterize the heterogeneity of the pore networks while the fourth variable, ${\hat{\tau }}_{i}$, describes the combined advective/dispersive motion of the solute particles.

Our goals in this study are: (i) to extract possible relationships between dispersivities and pore structure parameters, and, (ii) to relate the measured advection/dispersion properties to the statistical properties of the corresponding random variables ${\hat{r}}_{i}$, ${\hat{u}}_{i}$, ${\hat{q}}_{i}$ and ${\hat{D}}_{i}$. In particular, we wish to estimate the evolution of the third spatial moment of the solute plume with time and thus quantitatively test the Fickian character of the simulated transport.

## Numerical Procedures

We essentially followed the same procedures as *Bernabé et al*.^35^. However, there is one important difference, namely, we restricted solute transport to the network backbone. Owing to round-off numerical errors, solute particles in *Bernabé et al*.’s simulations had an extremely small but nevertheless non-zero probability to enter dangling pores (i.e., pores that do not belong to the backbone)^35^. Despite their rarity, these uncontrolled events significantly increased the ensemble statistical fluctuations of dispersivity and therefore the uncertainty of the results. Here, we preferred to avoid this problem completely by removing the dangling pores. Percolation models that focus on advective transport, also restrict solute motion to the backbone of the pore network^17,20^. Diffusion traps such as the dangling pores are often present in porous media, of course, and will be properly simulated in our future work (specific rules governing diffusion transport need to be devised). Details of the various stages of the numerical procedures are briefly described below.

### Construction of the network realizations

We constructed nominally isotropic networks of cylindrical pores using three different types of underlying three-dimensional lattices, simple cubic (SC), body-centered cubic (BCC) and face-centered cubic (FCC). The pore radii, *r*_*i*_, were assigned according to truncated log-uniform distributions such that *R*, the hydraulic radius of the network (i.e., two times the ratio of the total pore volume by the total pore surface area), had a fixed value. The log-uniform distribution is strongly skewed as often observed in rocks^35^. We used distributions with different coefficients of variation *CV* (i.e., standard deviation normalized to the mean). *R* and *CV* are related to the upper and lower limits of the distributions, *r*_max_ and *r*_min_, by

2 $$R=\frac{{r}_{{\rm{\min }}}+{r}_{\max }}{2}$$

3 $$CV=\sqrt{\frac{({r}_{\max }+{r}_{\min })\mathrm{ln}({r}_{\max }/{r}_{\min })}{2({r}_{\max }-{r}_{\min })}-1}$$

We also changed the connec**t**ivity of the networks by randomly selecting pores according to a probability 1 − *Z*/*Z*_max_ and setting their radii to zero. In the preceding rule, *Z* denotes the bond coordination number (i.e., mean number of bonds per node) and *Z*_max_ is the maximum possible bond coordination number in each lattice type (i.e., 6, 8 and 12 for SC, BCC and FCC, respectively). Note that the critical coordination number *Z*_c_ at the percolation threshold is nearly equal to 1.5 in three-dimensional lattices, including SC, BCC, FCC as well as Delaunay triangulation networks^38^. Hence, *Z*_c_ is an approximately universal parameter in the sense of percolation theory. It is well known that the ensemble statistical fluctuations in the network properties (e.g., permeability or dispersivity) are increasingly severe when the percolation threshold is approached. We only considered coordination numbers greater than 2.5 to avoid the additional complication of large ensemble fluctuations.

We determined the network backbone by identifying the nodes with a local coordination number of 1, removing the single pore attached to them and repeating the process until no new dead-end pores could be found. We verified that the backbone was complete by assigning a unit conductance value to all pores belonging to the presumed backbone and zero to the other ones, simulating electrical conduction through the resistor network thus formed, searching for bonds carrying zero current and removing them^39^. We found that the node exploration technique alone gave accurate results as long as coordination numbers *Z* > 2.5 were considered, but failed for networks closer to the percolation threshold. All results reported hereafter correspond to backbones of networks with *Z* > 2.5.

### Fluid flow simulation

To calculate the mean fluid velocity *u*_*i*_ in the pores we used the standard method of solving the linear system of Kirchoff equations (mass conservation) for the fluid pressure at the nodes and then applied Poiseuille equation to determine the flow rates *q*_*i*_ and mean velocities *u*_*i*_ = *q*_*i*_/(π*r*_*i*_^2^). For solving the Kirchoff system, we used the successive over-relaxation (SOR) iterative method, a technique especially well suited for partially connected networks (*Z* < *Z*_max_). In these calculations, we adjusted the global pressure gradient to produce a previously specified macro-scale fluid velocity *U* = ∑*r*_*i*_^2^*u*_*i*_/∑*r*_*i*_^2^, where the summations are effected on a network face normal to the flow.

### Motion of the solute particles

Our main assumption is that, at the scale of an individual pore, the solute particles obey Taylor dispersion. We simulate the combined advective-dispersive motion of a particle in a given pore by random drawing a realization of the transit time according to the cumulative distribution function given in equation 1. To save CPU time we calculated and stored digital representations (100 points) of the CDF’s for all pores in the network backbone using the local values of *r*_*i*_, *u*_*i*_ and *D*_*i*_. The digital CDF’s were then used whenever necessary to generate realizations of the transit times by the inverse transform method^35^.

A particle exiting a pore can enter into any of the connected pores, provided their fluid velocities are outwardly oriented (the particles are not allowed to travel against the local flow). To select the outwardly flowing pore into which the particle enters next, we used the perfect mixing rule, i.e., the next pore is randomly chosen with a probability proportional to its flow rate. The alternative rule (stream tube routing) can only be implemented in simple, effectively two-dimensional systems^40,41^. Moreover, it was previously found that the perfect mixing rule actually permits accurate simulation of longitudinal dispersion but tends to over-estimate transverse dispersion^28^. Therefore, although they are qualitatively consistent with experimental observations, our simulations of transverse dispersion may not be quantitatively meaningful. Thus, only longitudinal dispersion will be discussed in this paper.

### Method of moments

We injected the solute by introducing from 10000 to 50000 particles at time zero in pores located on the downstream face of the network corresponding to *X* = 0, where *X* denotes the nominal flow direction. The particles were randomly distributed according to a probability proportional to the cross-section area of the pores. This wide initial distribution of the particles in the transverse directions *Y* and *Z* facilitates fast sampling by the solute particles of the velocity field (complete velocity sampling is necessary to reach the asymptotic regime)^42^. We then propagated the particles through a large periodic array of identical copies of the current network realization and recorded their positions *X*_i_, *Y*_i_ and *Z*_i_ at different fixed times (for example, 5000, 10000, 20000, 40000, 60000, and 100000 s when *U* was set to 10^−4^ ms^−1^, corresponding to average traveled distances of 0.5, 1, 2, 4, 6 and 10 m). According to the method of moments^43^, the plume of particles at these different times can be characterized by the central moments, *M*_n_(*t*) = 〈(*X*_i_ − 〈*X*_i_〉)^n^〉, *K*_n_(*t*) = 〈(*Y*_i_ − 〈*Y*_i_〉)^n^〉 and *L*_n_(*t*) = 〈(*Z*_i_ − 〈*Z*_i_〉)^n^〉, where *n* is the order of the moment. Owing to the nominal isotropy of the network realizations, *K*_n_(*t*) and *L*_n_(*t*) are expected to be nearly equal. If the dispersion process is Fickian, the following relations must hold: 〈*X*_i_〉 ≈ *Ut*, 〈*Y*_i_〉 ≈ 〈*Z*_i_〉 ≈ 0, *M*_2_(*t*) = 2*D*_L_*t*, *K*_2_(*t*) ≈ *L*_2_(*t*) = 2*D*_T_*t* and *M*_3_(*t*) ≈ *K*_3_(*t*) ≈ *L*_3_(*t*) ≈ 0, where the constants *D*_L_ and *D*_T_ are the longitudinal and transverse macroscopic dispersion coefficient, respectively. Thus, the method of moments provides a convenient way to test whether or not the asymptotic regime is established (linear time dependence of the second moments) and to identify non-Fickian behavior (growing and non-zero third moments).

## Results

We considered ranges of the input parameters similar to those used in *Bernabé et al*.^35^: the network hydraulic radius *R* ranged from 20 to 100 × 10^−6^ m (the pivot, i.e., most frequent value, was 40 × 10^−6^ m), the pore radii coefficient of variation *CV* varied from 0.05 to 1.05, the mean coordination number *Z* from 2.5 to 12 and the macro-scale fluid velocity *U* from 10^−5^ to 10^−2^ ms^−1^ (the pivot was 10^−4^ ms^−1^). We investigated ratios of pore length to hydraulic radius *l*/*R* between 5 and 10 (the pivot was 7.5) and we used a single value for the coefficient of molecular diffusion (i.e., 10^−10^ m^2^ s^−1^). The velocity values were selected to insure that the simulated dispersion coefficients were proportional to *U*. They tend to be high compared to naturally occurring groundwater flow but still lie in the range corresponding to experimental or geotechnical applications.

### First and second central moments

We will first report the results concerning the first and second central moments of the solute plume and their evolution. Many of the following observations were already reported in *Bernabé et al*. and therefore do not need to be illustrated by specific figures^35^.

**Figure 1:**
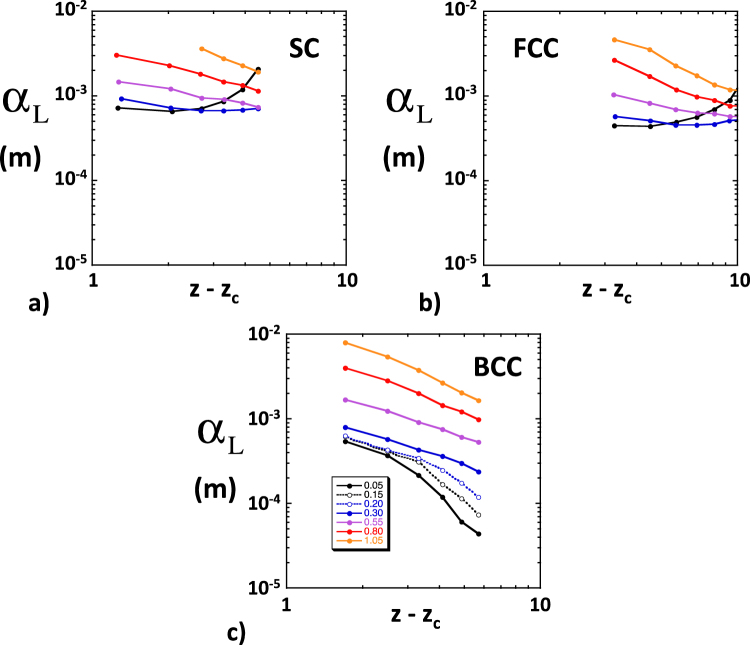
Simulated longitudinal dispersivity as a function *Z* − *Z*_c_. These simulations were run in (**a**) SC, (**b**) FCC and (**c**) BCC network realizations. In these examples, we used 10000 particles, *U* = 10^−4^ ms^−1^, *R* = 40 × 10^−6^ m, *l* = 300 × 10^−6^ m and 7 different coefficients of variations *CV*, namely, 0.05 (solid black), 0.15 (dotted black), 0.20 (dotted blue), 0.30 (solid blue), 0.55 (purple), 0.80 (red) and 1.05 (orange).

**Figure 2:**
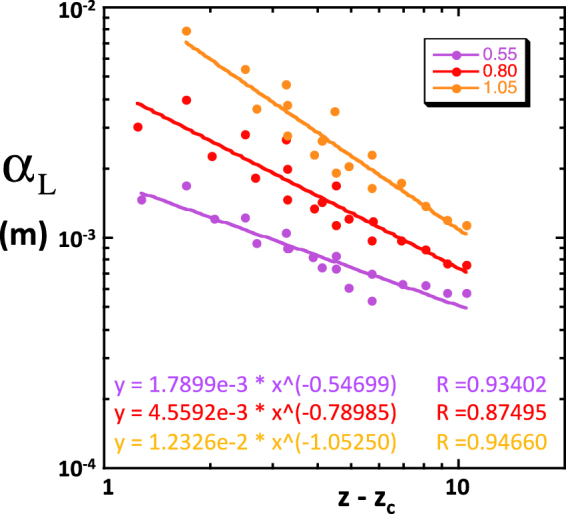
Approximately “universal” power law relationships of the simulated dispersivities with *Z* − *Z*_c_ in networks realizations with *CV* ≥ 0.55 (indicated using the same colors as in Fig. 1). The clusters of data-points include SC, BCC and FCC simulations. The data-point scatter helps visualize the statistical uncertainty associated with each value of *CV*. The best fitting equations are indicated in matching colors. The simulations corresponding to *CV* = 0.05 and 0.30 are omitted because they produce results that depend on lattice type (see text).

**Figure 3:**
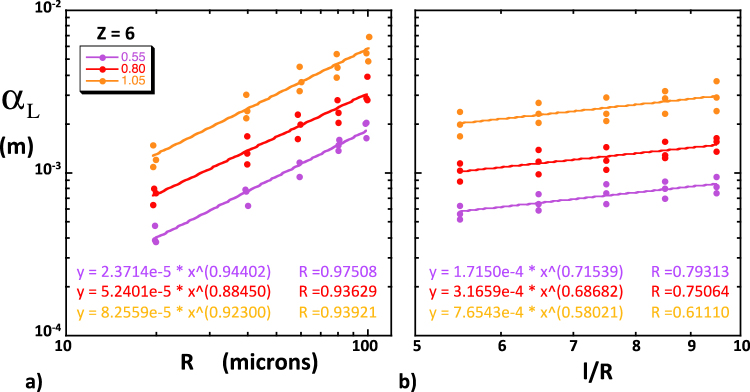
Approximate power law dependence of the simulated longitudinal dispersivities on (**a**) the network hydraulic radius *R* and (**b**) the bond length-to-radius ratio *l*/*R*. These simulations correspond to BCC networks realizations with *CV* ≥ 0.55 (indicated using the same colors as in Fig. 1). These simulations were performed with 10000 particles, *Z* = 6, *U* = 10^−4^ ms^−1^, *l*/*R* = 7.5 (**a**) and *R* = 40 × 10^−6^ m (**b**). The best fitting equations are indicated in matching colors. The simulations corresponding to *CV* = 0.05 and 0.30 are omitted because they produce results that depend on lattice type (see text).

- In all cases, the center of mass of the solute plume (first moment) traveled downstream at a constant velocity that was very nearly equal to the imposed fluid velocity *U*, indicating that the solute particles sampled the entire set of local velocities characterizing the flow field. This good agreement was probably due to our restricting transport to the network backbone.
- We always observed a linear growth of the second moments *M*_2_(*t*) and *K*_2_(*t*) ≈ *L*_2_(*t*), indicating that the asymptotic regime was reached earlier than the shortest time analyzed (5000 s for *U* = 10^−4^ ms^−1^). We could therefore unambiguously define and measure *D*_L_ and *D*_T_ in all simulations. The shortness of the pre-asymptotic regime was due to the periodic structure used here^44^. Indeed, the particles travelled more than 200 network lengths in the shortest simulated time analyzed, largely enough for the set of particles to sample the velocity field completely and to reach the asymptotic regime.
- The simulated longitudinal dispersion coefficient was more than one order of magnitude larger than the transverse coefficient, in general agreement with experimental observations^18,45^. In equivalent conditions of *U*, *R*, *CV* and *Z*, the dispersion coefficients simulated here were smaller by a factor up to 2~3 than the coefficients reported by *Bernabé et al*.^35^, most likely because they did not restrict solute transport to the network backbone as we did.
- The simulated coefficients *D*_L_ and *D*_T_ were found linearly related to *U*. This result is consistent with compilations of experimental values of *D*_L_ showing a roughly linear relationship of *D*_L_ and the Péclet number, Pe = *lU*/*D*_m_, for Pe greater than ten^45^. Here, the simulations were run in a range of Pe between 30 and 30000. This result also enables us to report our results in terms of the dispersivities, α_L_ = *D*_L_/*U* and α_T_ = *D*_T_/*U*.
- The dependence of the simulated α_L_ and α_T_ on the coordination number difference (*Z* − *Z*_c_) was different in SC, BCC and FCC networks (see examples for α_L_ in Fig. 1, corresponding to 10000 particles, *U* = 10^−4^ ms^−1^, *R* = 40 × 10^−6^ m and *l* = 300 × 10^−6^ m). Thus, our network simulations of dispersion do not behave like similarly implemented network simulations of permeability or electrical conductivity, which consistently demonstrated independence on lattice type. We note, however, that the discrepancies between SC, BCC and FCC primarily occurred for low values of the heterogeneity measure *CV* and high coordination numbers. For example, the nearly homogeneous SC and FCC networks (*CV* = 0.05) displayed an uncharacteristic increase of the simulated α_L_ with increasing *Z*, opposite to the typical trend of decreasing dispersivity with increasing connectivity seen in all networks with *CV* > 0.3. The lattice-dependent behavior of the nearly homogeneous SC and FCC networks can be attributed to the presence of bonds perpendicular to the macroscopic pressure gradient. These bonds carry negligible flow and, hence, act as traps for the few solute particles entering them^35^. On the other hand, all bonds in the BCC lattice are equally inclined and are therefore unlikely to trap particles. Yet, the sharp decrease of α_L_ with increasing *Z* − *Z*_c_ observed in BCC networks with CV < 0.3 may also be singular (see Fig. 1c). However, we found that a sufficient amount of disorder (e.g., introduced by increasing *CV* beyond 0.3) produced lattice-independence (i.e., approximate universality in the sense of percolation theory). Indeed, when all SC, BCC and FCC data corresponding to *CV* ≥ 0.55 are mixed together, we observe relatively well-defined power laws α_L_ ∝ (*Z* − *Z*_c_)^−*m*^, where, moreover, the exponent *m* is very nearly equal to *CV* (Fig. 2). Other power laws, α_L_ ∝ *R*^β^ and α_L_ ∝ (*l*/*R*)^γ^, were observed (Fig. 3; BCC simulations with 10000 particles, *Z* = 6, *CV* ≥ 0.55 and *U* = 10^−4^ ms^−1^; *l*/*R* = 7.5 in Fig. 3a yielded β ~ 0.9; *R* = 40 × 10^−6^ m in Fig. 3b produced small variations of γ between 0.6 and 0.7).

### Third moments

**Figure 4:**
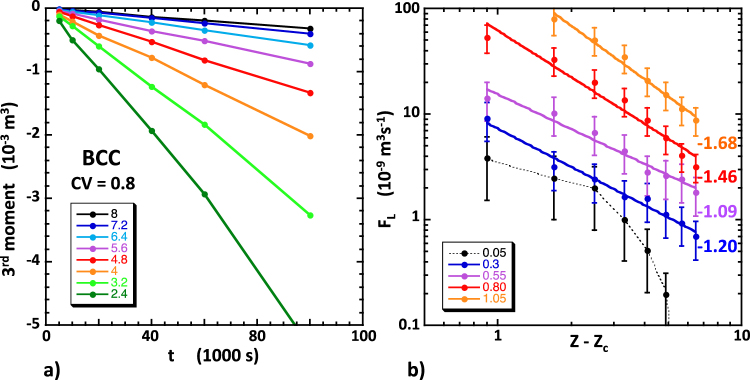
(**a**) Examples of the evolution with time of the simulated third moments *M*_3_(*t*). The data shown are averages of pairs of simulations in identical conditions (BCC, 50000 particles, *CV* = 0.80, *U* = 10^−4^ ms^−1^ and *Z* between 3.2 and 8 as indicated by the colored symbols). (**b**) Relationship of the time rate coefficient *F*_L_ and *Z* − *Z*_c_. For *CV* ≥ 0.3 the data obey approximate power laws, the exponents of which are indicated in matching colors. For *CV* = 0.05 a power law is not observed. The error bars represent the ensemble statistical fluctuations expected in these simulations.

We performed additional simulations, during which we recorded the third moments *M*_3_(*t*), *K*_3_(*t*) and *L*_3_(*t*) at the same fixed times as mentioned earlier. It is well known that simulated moments exhibit ensemble statistical fluctuations that grow with the moment order. Thus, to ensure statistical stability and a sufficient level of accuracy, we increased the number of particles to 50000. In all simulations, the longitudinal moment *M*_3_(*t*) had negative values, corresponding to a solute plume shaped like a water-drop with a relatively long trailing wake. We also observed that the absolute value of *M*_3_(*t*) increased linearly with increasing time according to |*M*_3_(*t*)| ≈ *F*_L_ *t* (see Fig. 4a, showing the ensemble-averaged results of five BCC simulations, with 10000 to 50000 particles, *CV* = 0.80, *U* = 10^−4^ ms^−1^, *R* = 40 × 10^−6^ m and *l* = 300 × 10^−6^ m). Thus, we can conclude that the asymptotic regime was established simultaneously for the first, second and third moments and that long-term macroscopic dispersion was intrinsically non-Fickian in our network simulations. Furthermore, we found that, except for *CV* = 0.05, the proportionality coefficient *F*_L_ had an approximate power law dependence on *Z* − *Z*_c_ (Fig. 4b, showing ensemble-averaged values over 5 simulations). The exponents of these power laws ranged from ~ −1 to ~ −2 and seemed to decrease with increasing *CV*. The specific values of the exponent observed may not be accurate, however, owing to the substantial statistical uncertainties expected. The transverse moments *K*_3_(*t*) and *L*_3_(*t*) were negligible compared to *M*_3_(*t*) and fluctuated irregularly both in magnitude and sign.

### Statistics of pore-scale fluid velocities, flow rates and Taylor dispersion coefficients

The results described above were produced in each network realization by the variations in the local pore-scale hydrodynamic conditions, i.e., the values of the flow rates *q*_*i*_, Eulerian mean fluid velocities *u*_*i*_ and Taylor dispersion coefficient *D*_*i*_. To quantify these variations, we recorded the complete sets of *q*_*i*_, *u*_*i*_ and *D*_*i*_ values produced in simulations of fluid flow through BCC network realizations (with *CV* = 0.05, 0.55 and 1.05, *Z* = 2.8, 3.2, 4.0, 4.8, 6.4 and 8.0, *U* = 10^−4^ ms^−1^, *R* = 40 × 10^−6^ m and *l* = 300 × 10^−6^ m). To facilitate the description of these data sets, we normalized the parameters with respect to the values expected in an exactly homogeneous, fully connected BCC network, namely, *r*_i_* = *r*_*i*_/*R*, *u*_i_* = *u*_*i*_/*U*, *q*_i_* = *q*_*i*_/*q*_0_ (with *q*_0_ = *U*π*R*^2^) and *D*_i_* = *D*_*i*_/*D*_0_ (with *D*_0_ = *D*_m_ + *R*^2^*U*^2^/(48*D*_m_)). It is not necessary to discuss the pore radii distributions since they were directly assigned log-uniform distributions obeying equations 2 and 3. It is worth noting, however, that the mean pore radius 〈*r*_*i*_〉 is not equal to the hydraulic radius *R* but to *R*/(1 + *CV*^2^).

#### Pore-scale fluid velocities

**Figure 5:**
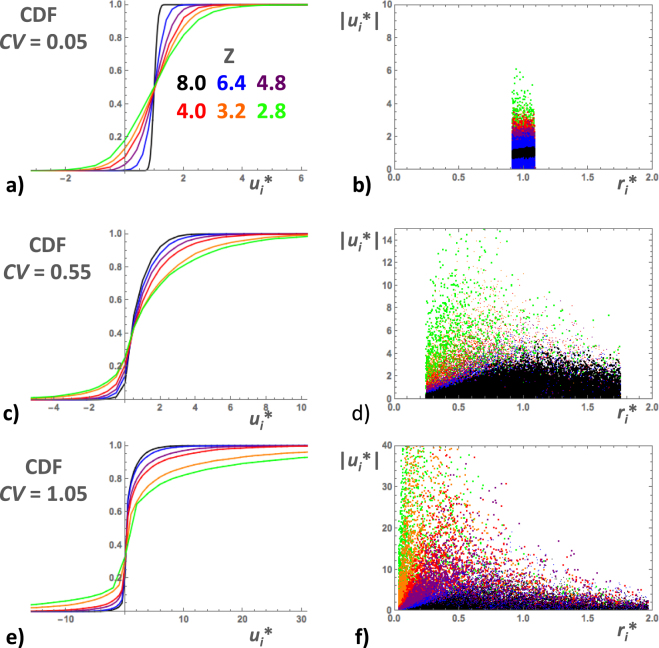
Examples of the cumulative distribution function (CDF) of *u*_i_* in BCC simulations with *CV* = 0.05 (**a**), 0.55 (**c**) and 1.05 (**e**). In each diagram, the CDF’s corresponding to different coordination numbers (*Z* = 8.0, 6.4, 4.8, 4.0, 3.2 and 2.8) are represented by colored lines as indicated in the inset. Scatterplot of |*u*_i_*| versus *r*_i_* for the same simulations, *CV* = 0.05 (**b**), 0.55 (**d**) and 1.05 (**f)**. Layers of colored data-points are superposed with the ones corresponding to *Z* = 8 on top and *Z* = 2.8 at the bottom. (See text for more details).

**Figure 6:**
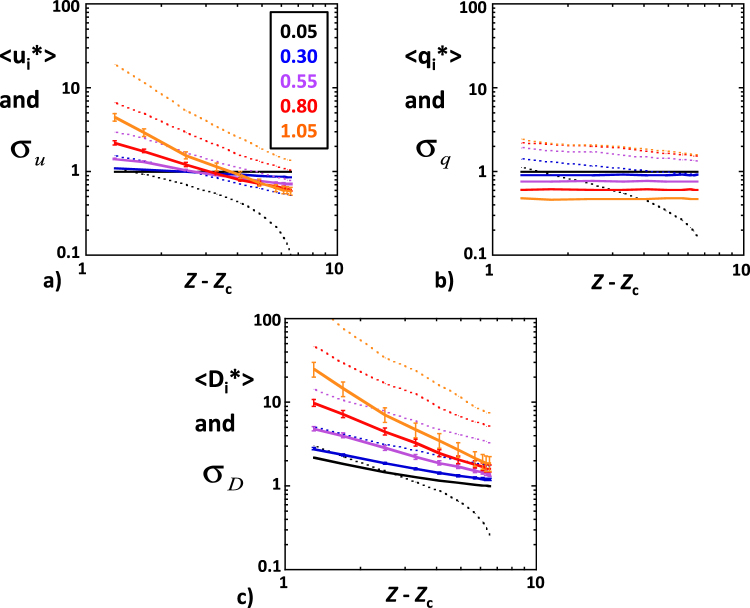
Means (solid lines) and standard deviations (dotted lines) of the distributions of the normalized pore velocities *u*_i_* (**a**), flow rates *q*_i_* (**b**) and Taylor dispersion coefficients *D*_i_* (**c**) as functions of *Z* − *Z*_c_ from BCC simulations with *CV* values indicated in the inset.

The simulated values of *u*_i_* were nearly normally distributed (symmetric CDF’s, actually well fitted by normal distribution CDF’s; see Fig. 5a) in network realizations with very narrow radius distributions (*CV* = 0.05) and became more and more skewed as *CV* increased (see Fig. 5c and e). Negative velocities occurred in all cases, except the most homogeneous one, i.e., *CV* = 0.05 and *Z* = 8 (Fig. 5a). The proportion of negative velocities increased substantially with increasing *CV* and decreasing *Z* − *Z*_c_. The local fluid velocities were not correlated to the local pore radii as demonstrated by scatter-plots of |*u*_i_*| versus *r*_i_* (see Fig. 5b,d,f). The shape of the data-point clusters changed significantly with *CV* and *Z* (horizontal bands for *CV* = 0.05, Fig. 5b, horizontal bands transforming into increasingly wide sectors for *CV* ≥ 0.55, Fig. 5d,f). Note that the data-points in Fig. 5b,d,f are arranged in superposed layers, so that the data-points corresponding to low *CV*’s mask the high *CV* ones. The average velocities 〈*u*_i_*〉 of the network realizations approximately followed power laws, 〈*u*_i_*〉∝ (*Z* − *Z*_c_)^−*b*^, where the exponent *b* increased from nearly zero to 1.2 with increasing *CV* (Fig. 6a). The velocity standard deviation σ_u_ increased with increasing *CV* and, except for *CV* = 0.05, also obeyed approximate power laws, σ_u_ ∝ (*Z* − *Z*_c_)^−*c*^, where the exponent *c* increased from about 0.7 to 1.6 with increasing *CV* (Fig. 6a). Qualitatively similar results were obtained in *Vasilyev et al*.^34^.

#### Pore-scale flow rates

**Figure 7:**
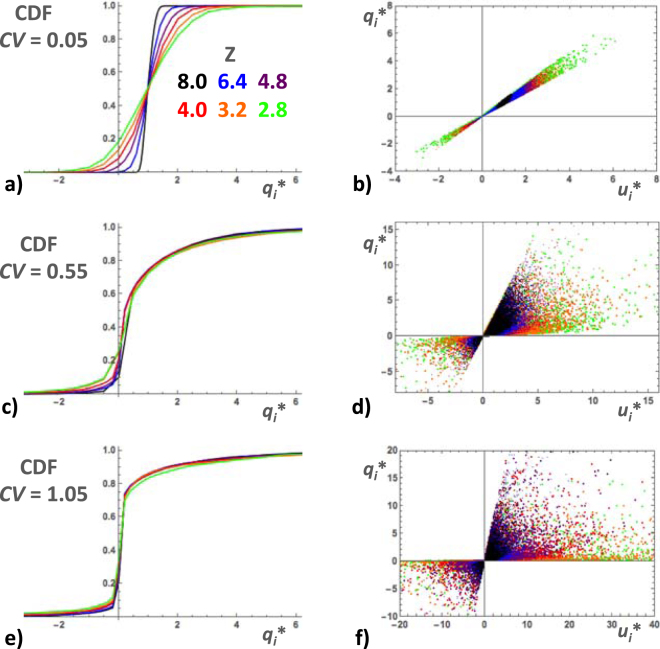
Examples of the cumulative distribution function of *q*_i_* in BCC simulations with *CV* = 0.05 (**a**), 0.55 (**c**) and 1.05 (**e**). In each diagram, the CDF’s corresponding to different coordination numbers (*Z* = 8.0, 6.4, 4.8, 4.0, 3.2 and 2.8) are represented by colored lines as indicated in the inset. Scatterplot of *q*_i_* versus *u*_i_* for the same simulations, *CV* = 0.05 (**b**), 0.55 (**d)** and 1.05 (**f**). Layers of colored data-points are superposed with the ones corresponding to *Z* = 8 on top and *Z* = 2.8 at the bottom. (See text for more details).

The simulated values of *q*_i_* had similar characteristics as *u*_i_*, namely, nearly normal distribution for *CV* = 0.05 (Fig. 7a), becoming more and more skewed at increasing *CV*’s (Fig. 7c,e). The main difference was that the effect of *Z* was considerably reduced in high *CV* simulations. Scatter-plots of *q*_i_* versus *u*_i_* show that fluid velocities and flow rates were relatively well correlated in simulations corresponding to *CV* = 0.05 (Fig. 7b) but became increasingly uncorrelated as pore radius heterogeneity was increased. The data-point clusters formed widening sectors, with negative *q*_i_* automatically corresponding to negative *u*_i_* (Fig. 7bdf). The average pore-scale flow rate 〈*q*_i_*〉 was independent of *Z* − *Z*_c_ and decreased increasing *CV* (Fig. 6b). The flow rate standard deviation σ_q_ moderately increased with increasing *CV*, while approximately following a power law, σ_q_ ∝ (*Z* − *Z*_c_)^−1/4^, again with exception of the simulations with *CV* = 0.05 (Fig. 6b).

#### Taylor dispersion coefficients

**Figure 8:**
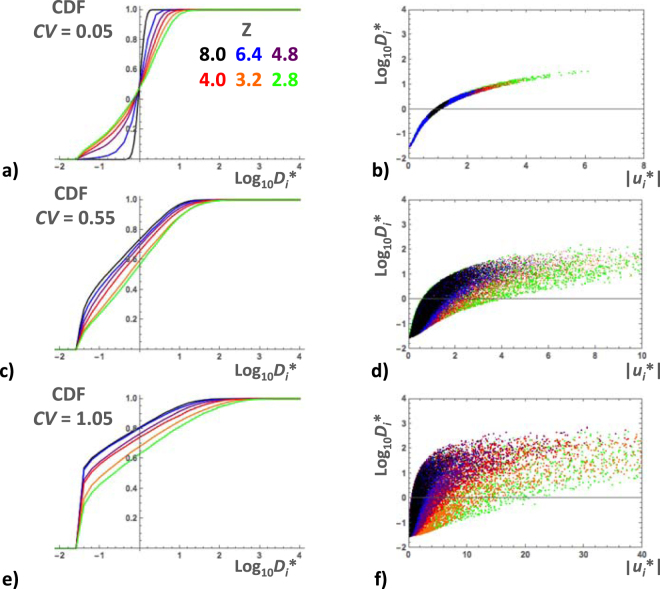
Examples of the cumulative distribution function of Log_10_*D*_i_* in BCC simulations with *CV* = 0.05 (**a**), 0.55 (**c**) and 1.05 (**e**). In each diagram, the CDF’s corresponding to different coordination numbers (*Z* = 8.0, 6.4, 4.8, 4.0, 3.2 and 2.8) are represented by colored lines as indicated in the inset. Scatterplot of Log_10_*D*_i_* versus |*u*_i_*| for the same simulations, *CV* = 0.05 (**b**), 0.55 (**d**) and 1.05 (**f**). Layers of colored data-points are superposed with the ones corresponding to *Z* = 8 on top and *Z* = 2.8 at the bottom. (See text for more details).

Since it is a function of the product *r*_i_*^2^*u*_i_*^2^, the pore-scale Taylor dispersion coefficient *D*_i_* varies substantially more than either *r*_i_* and *u*_i_*. Hence, we considered Log_10_*D*_i_* rather than *D*_i_* itself. The CDF’s of Log_10_*D*_i_* were marginally more skewed than the normal distribution CDF in simulations with *CV* = 0.05 (Fig. 8a) and evolved with increasing *CV* towards uniform distributions, truncated at the low end because *D*_i_* reaches the minimum value of *D*_m_/*D*_0_ when the local fluid velocity becomes very low (Fig. 8e). In simulations with *CV* = 0.05, *r*_*i*_ had a very narrow range of variation around *R* and, therefore, *D*_i_* was nearly equal to *u*_i_*^2^ (except when *u*_*i*_ was very near zero), thus producing parabolic data-point clusters (Fig. 8b). The data-point clusters became gradually more dispersed as *CV* was increased (Fig. 8d,f). The average local Taylor dispersion coefficient 〈*D*_i_*〉 increased strongly with increasing *CV* and decreasing *Z* − *Z*_c_ (Fig. 6c). Its minimum value, 〈*D*_i_*〉 ≈ 1, was reached for *CV* = 0.05 and *Z* = 8. As before, 〈*D*_i_*〉 and the standard deviation σ_D_ displayed approximate power laws, 〈*D*_i_*〉 ∝ (*Z* − *Z*_c_)^−*b*^ and σ_D_ ∝ (*Z* − *Z*_c_)^−*c*^, with the exponents *b* and *c* increasing from about 0.5 and 0.7 to 1.6 and 1.8, respectively (Fig. 6c).

## Discussion

Our results confirm that increasing pore-size heterogeneity and/or decreasing pore connectivity enhance dispersivities simulated in pore networks^35^. Moreover, the newly observed growth with time of the third central moment suggests that the simulated dispersion process is intrinsically non-Fickian. Importantly, non-Fickianity in our network simulations does not seem to be related to a transitory transport regime because the constantly observed linear growth with time of the central moments indicated a fully established asymptotic regime in all cases. Because our simulations were performed on network backbones and all dangling pores with zero fluid velocity were removed, the non-Fickian behavior observed here cannot be attributed to the existence of diffusion traps (i.e., stagnant pores) as assumed in some theoretical models^4,11,18^ and sometimes experimentally observed^6,12^. Certain percolation models do not include stagnant pores and relate solute dispersion to the tortuosity of the critical paths^17,20^. Diffusion traps also tend to retard solute transport, so that the velocity of the solute center-of-mass is lower than the mean fluid velocity. Retardation effects were not observed in our backbone-restricted simulations since the velocity of the solute center-of-mass was always nearly equal to the imposed mean fluid velocity.

Thus, the results summarized above were produced by the hydrodynamic random variables, ${\widehat{u}}_{i}$, ${\hat{D}}_{i}$, ${\hat{q}}_{i}$ and ${\hat{\tau }}_{i}$, and their response to different distributions of the geometric random variable, ${\hat{r}}_{i}$ or, or, in other words, to changes in pore-size heterogeneity and pore connectivity. Indeed, the behavior of 〈*u*_i_*〉, 〈*D*_i_*〉, σ_u_ and σ_D_ with increasing *CV* and/or decreasing *Z* − *Z*_c_ was qualitatively similar to that of the dispersivity α_L_. Remarkably, examinations of ${\widehat{u}}_{i}$, ${\hat{D}}_{i}$ and ${\hat{q}}_{i}$ showed that they were influenced differently by *CV* and *Z* − *Z*_c_. The difference was particularly clear for ${\hat{q}}_{i}$, for which 〈*q*_i_*〉 decreased with increasing *CV* but was unaffected by *Z* − *Z*_c_. We also note a stronger differentiation of the effects of *CV* and *Z* − *Z*_c_ in conditions of near pore-size homogeneity (*CV* ≤ 0.3). For example, cross-plots of α_L_ against σ_u_ or σ_D_ at constant values of *CV* reveal approximate power law relationships, whose exponents moderately vary for *CV* above 0.3 but sharply increase at low *CV* values. Thus, trying to identify general relationships of α_L_ with σ_u_, σ_D_ or other statistical characteristics of ${\widehat{u}}_{i}$, ${\hat{D}}_{i}$and ${\hat{q}}_{i}$ is probably not an effective approach. It is more fruitful to focus on the transit time random variable, ${\hat{\tau }}_{i}$^10–12,23,24^.

### Lagrangian transit times and velocities

One particularly useful approach is to consider ${\hat{\tau }}_{i}$ from a Lagrangian viewpoint^28^. We ran additional BCC simulations, during which we recorded the motion of individual particles, i.e., we saved the values of 150,000 successive transit times τ_*i*_ experienced by each particle along its particular trajectory. The transit times ranged over more than 6 orders of magnitude and it was, therefore, convenient to consider their decimal logs hereafter. Since the local fluid velocities *u*_*i*_ can be positive or negative, we calculated two Lagrangian transit-time probabilities *P*^+^(τ) = *P*(+*l*, τ) and *P*^−^(τ) = *P*(−*l*, τ) by forming two separate lists of Log_10_(τ_*i*_) corresponding to forward and backward advective motion. We then performed bin counts of the elements of these lists (we used a bin width of a half-order of magnitude as a compromise between resolution and uncertainty). The probabilities *P*^+^(τ) and *P*^−^(τ) were estimated as the ratios *N*^+^(τ)/*N* and *N*^−^(τ)/*N* of the numbers of forward and backward transit times present in the bin containing τ by the total number of pore transitions (i.e., 150,000). The results were ensemble-averaged over 4 realizations and 5 particles per realization.

**Figure 9:**
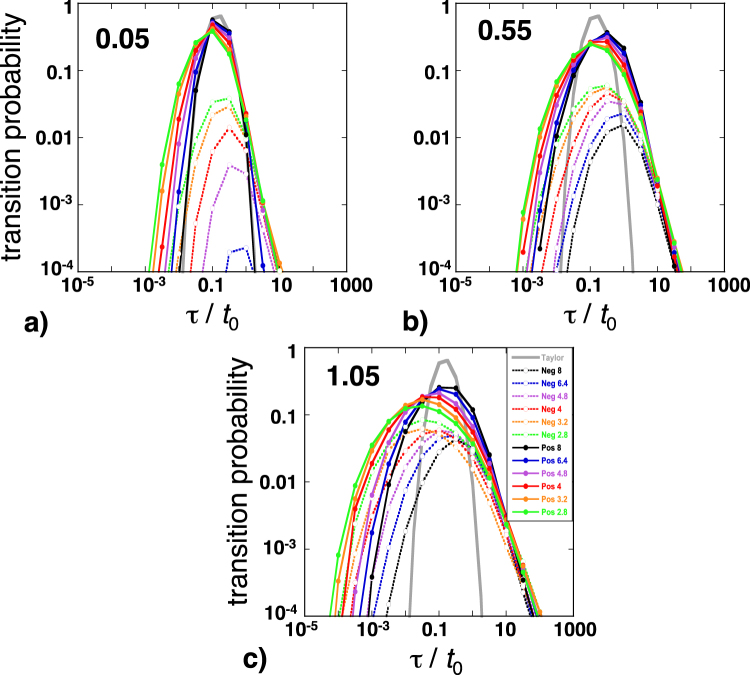
Transit time probabilities *P*^+^(τ/*t*_0_) (solid lines) and *P*^−^(τ/*t*_0_) (dotted lines) for BCC simulations with *CV* = 0.05 (**a**), 0.55 (**b**) and 1.05 (**c)**. The curves corresponding to different values of *Z* are represented in colors as indicated in the inset. The thick grey curve in each diagram represents the theoretical transit time probabilities inferred from the Taylor dispersion transit time CDF (equation 1) with a velocity *U* and a dispersion coefficient *D*_0_. (See text for more details).

We observed that the arch-like curves of *P*^+^ and *P*^−^ versus the normalized transit time τ/*t*_0_ (with *t*_0_ = *l*/*U*) became broader with increasing *CV* and decreasing *Z* − *Z*_c_, in agreement with the growth of the simulated dispersivity in the same conditions (Fig. 9). As logically expected, the *P*^+^(τ/*t*_0_) curve for the most homogeneous network (*CV* = 0.05 and *Z* = 8) coincided almost exactly with the theoretical curve inferred from the Taylor dispersion transit-time CDF (equation 1) with a velocity *U* and a dispersion coefficient *D*_0_ (note that the *P*^−^(τ/*t*_0_) curve did not exist in this case; Fig. 9). Most importantly, the low- and high-τ flanks of the *P*^+^(τ/*t*_0_) and *P*^−^(τ/*t*_0_) curves responded differently to changes in *CV* and *Z* − *Z*_c_. The low-τ branches tended to shift horizontally with variations of both *CV* and *Z* − *Z*_c_ while the high-τ branches of both *P*^+^(τ/*t*_0_) and *P*^−^(τ/*t*_0_) approximately fell on top of a single power law, *P*^+^(τ/*t*_0_) ≈ *P*^−^(τ/*t*_0_) ∝ (τ/*t*_0_)^−*f*^, depending on *CV* but not on *Z* − *Z*_c_ (Fig. 9). Moreover, the exponent *f* decreased with increasing pore-size heterogeneity (namely, *f* ≈ 2.5, 2 and 1.5 for *CV* = 0.05, 0.55 and 1.05, respectively). Actually, a power law form of the transit-time probability function is often associated with non-Fickian dispersion^10–12,28^ and the non-Fickian character is enhanced when the exponent *f* decreases. Low values of *f* around 1.5 are usually associated with the presence of stagnant micro-porosity^12^ but were obtained here by simply increasing the width of the pore radius distribution. We can also infer from our simulations that the non-Fickian behavior observed was not solely due to the high-τ power laws discussed above, but was also affected by the low-τ branches of the *P*^+^(τ/*t*_0_) and *P*^−^(τ/*t*_0_) curves. Indeed, if the high-τ power laws were the sole contributors to non-Fickianity, we should not observe any effect of pore connectivity *Z* − *Z*_c_ on the 3^rd^ moment coefficient *F*_L_ and its growth, contrary to the results shown in Fig. 4b. We note also that, owing to the observed agreement of the velocity of the solute center-of-mass with *U*, any elongation of the high-τ tail must be balanced by a change of the low-τ leading front. Elongation of the leading front is not possible since the velocity of individual solute particles has a finite upper limit. Examination of examples of solute plumes suggests that the leading fronts tended to become steeper with time, although we were not able to quantify this effect accurately. Indeed, significant differences of breakthrough curves with the best-fit ADE solutions have been experimentally observed at both early and late times^10,11^, suggesting that the low-τ leading front does indeed contribute to non-Fickian dispersion in real materials. We speculate that the large variability of the local Taylor dispersion coefficient (about 6 orders of magnitude for *CV* = 1.05, from values close to *D*_m_ to nearly 1000 *D*_0_) is one of the main factors producing this behavior. The variability of ${\hat{D}}_{i}$ is fundamentally caused by its dependence on the squares of ${\hat{u}}_{i}$ and ${\hat{r}}_{i}$, a functional form that should remain valid even in pores with irregular cross-sections^36,37^. The situation is very different in typical continuum models, for which the small-scale dispersion process is usually assumed constant and assigned a relatively small magnitude^8,42^.

One important feature of the network flow fields simulated here is the presence of backward flowing bonds. Backward pore transitions are not explicitly excluded in models such as the continuous time random walk approach of *Berkowitz et al*.^11^ but rarely included although negative fluid velocities have indeed been observed in simulations of flow through random packs of spheres and porous rocks^46^. Negative *u*_*i*_ were, of course, not the majority in our simulations and did not form long connected chains, thus precluding long-range backward advection (their effect is to increase the tortuosity of the flow paths^47^; see also *Hunt and Skinner*^17^ and *Hunt and Sahimi*^20^). Consequently, the *P*^−^(τ/*t*_0_) curves were always substantially lower than the *P*^+^(τ/*t*_0_) curves (Fig. 9). For a given simulation, the probability of a backward pore transition is given by:

4 $${p}^{-}=\sum {N}^{-}({\tau }_{i})/\sum ({N}^{+}({\tau }_{i})+{N}^{-}({\tau }_{i}))$$

We found that *p*^−^ grew from a minimum of exactly zero for the most homogeneous networks (*CV* = 0.05 and *Z* = 8) to a maximum of 30% for the most heterogeneous (*CV* = 1.05 and *Z* = 2.8) and was well related to the simulated dispersivities by α_L_ ≈ 1.7 × 10^−3^ Exp[14 *p*^−^].

**Figure 10:**
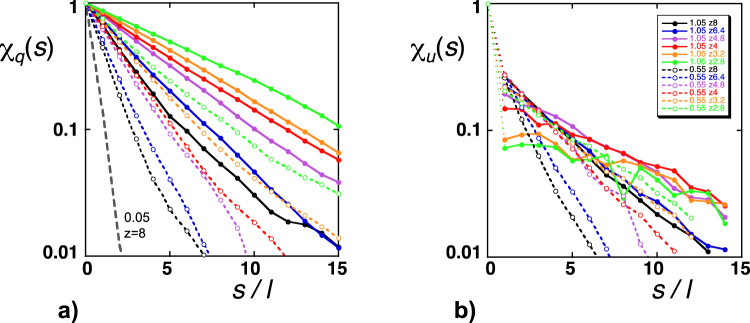
The autocorrelation functions, (**a**) χ_*q*_(*s*) and (**b**) χ_*u*_(*s*), as functions of the normalized separation distance *s*/*l*. These examples correspond to BCC simulations with *CV* = 0.05 (thick grey dashed line), 0.55 (dotted lines) and 1.05 (solid lines), and to different values of *Z* as indicated in the inset. The nugget effect characteristic of the χ_*u*_(*s*) curves is made more visible by using very thin lines at the beginning of the curves (see text for more details).

Another issue is that, owing to mass conservation, the sets of Lagrangian fluid velocities experienced by a solute particle have a correlated structure that can significantly affect macroscopic dispersion^28^. To estimate this effect we performed further BCC simulations, during which we recorded 15,000 pore velocities *u*_*i*_ and flow rates *q*_*i*_ successively encountered by individual particles along their trajectories (here, positive and negative velocities were recorded in the same list). We then calculated the autocorrelation functions, χ_*u*_(*s*) = Corr[*u*_*i*_(*x* + *s*), *u*_*i*_(*x*)] and χ_*q*_(*s*) = Corr[*q*_*i*_(*x* + *s*), *q*_*i*_(*x*)], for each set of *u*_*i*_ and *q*_*i*_ data (*x* is the longitudinal coordinate of the particle and *s* the separation distance). The results were ensemble-averaged over 4 realizations and 10 particles per realization. In the simulations with *CV* > 0.05, the flow rate autocorrelation functions approximately obeyed the exponential relationship, χ_*q*_(*s*/*l*) ≈ Exp[−(*s*/*l*)/*s*_*q*_] (Fig. 10a). The estimated correlation length *s*_*q*_ increased with increasing *CV* and decreasing *Z* − *Z*_c_ (namely, from 1.3 to 4.1 for *CV* = 0.55 and from 2.6 to 6.8 for *CV* = 1.05, with *Z* dropping from 8 to 2.8). A similar trend was observed in *Kang et al*.^28^. The pore velocity autocorrelation functions can be roughly described as a combination of exponential decay and nugget effect (sudden drop at the origin), χ_*u*_(*s*/*l*) ≈ χ_0_ Exp[−(*s*/*l*)/*s*_*u*_] (Fig. 10b). The nugget effect arises because *u*_*i*_, unlike *q*_*i*_, is not directly controlled by mass conservation and its magnitude (measured by 1 − χ_0_) reflects the de-correlating effect of random variations in pore radius. We found that 1 − χ_0_ was rather variable and tended to increase with increasing *CV* (namely, from 0.63 ± 0.11 to 0.79 ± 0.11 for *CV* = 0.55 and 1.05, respectively). The correlation length *s*_*u*_ was longer than *s*_*q*_ (namely, from 1.8 to 4.9 for *CV* = 0.55 and from 3.8 to 10.3 for *CV* = 1.05). These values are significantly greater than those reported in *Kang et al*.^28^. It must be noted that the description above did not apply to the simulations with the narrowest pore radius distributions, again indicating that nearly homogeneous BCC simulations have a singular behavior, probably not generalizable to porous rocks. Indeed, χ_*q*_(*s*/*l*) and χ_*u*_(*s*/*l*) in simulations with *CV* = 0.05 and *Z* < 8 showed fast decaying oscillations about zero (for *Z* = 8 the autocorrelation functions decayed to zero extremely fast, with no oscillations). The separation *s*/*l* corresponding to the first negative oscillation moved from 1 for *Z* = 6.4 to greater values at decreasing coordination numbers. To understand the origin of this behavior, we consider a perfectly homogeneous network (*CV* = 0) from which a single bond was removed. The flow field must be uniform everywhere in this network, except in the immediate vicinity of the missing pore, effectively an obstacle around which the fluid must revolve. We can visualize the flow field by idealizing the missing pore obstacle as a sphere. It becomes clear then that the fluid velocities normal to the upstream/downstream poles must be reduced while the velocities tangent to the equator are amplified. This alternated velocity structure produces anti-correlation of the fluid velocity at separations comparable to the diameter of the obstacle, similar to the negative oscillations displayed by χ_*q*_(*s*/*l*) and χ_*u*_(*s*/*l*) for dilute distributions of missing pores (high coordination numbers). When *Z* decreases, interactions between missing pores become more frequent and gradually dampen the oscillations of χ_*q*_(*s*/*l*) and χ_*u*_(*s*/*l*).

### Concluding remarks

Our numerical simulations are based on a physically based description of the pore-scale transport processes. Poiseuille law is used to determine the mean fluid velocity and Taylor dispersion to evaluate the distribution of transit times in individual pores. These rules include complex interactions among the geometric and hydrodynamic variables involved. The upside of this approach is that we can introduce very broad/skewed pore radius distributions and vary the pore connectivity. The downsides are the idealized pore geometry, the periodicity of the medium (preventing the occurrence of long transients), the perfect mixing rule (limiting the accuracy of simulated transverse transport) and the restriction of simulated transport to the backbone (excluding diffusion traps).

Studies such as *Kang et al*.^28^, or, *Bijeljic et al*.^25^ and *Bijeljic et al*.^26^ have strong similarities with ours but some differences arise owing to the different scales they used for the description of the transport processes. *Kang et al*.^28^ effectively worked at the continuum scale. They discretized the porous continuum into a two-dimensional square lattice, the elements of which obeyed Darcy’s law. They assumed that permeability varied spatially while porosity remained constant. As a consequence, the mean fluid velocity in any lattice bond was proportional to the flow rate and they obtained velocity autocorrelation functions (shown in their Fig. 8b) similar to our exponential χ_*q*_ curves (Fig. 10a). We also note the qualitative resemblance of *Kang et al*.’s Lagrangian transit-time distributions (in their Fig. 8a) to ours (Fig. 9). Quantitative differences (e.g., lower correlation length *s*_*q*_ of the Lagrangian velocities and exponent *f* of the transit-time distributions) had multiple causes such as differences in the heterogeneity levels considered, two-dimensional lattices or their assumption of purely advective transport in the lattice bonds. *Kang et al*.’s continuum approach does not allow explicit investigation of the effect of pore connectivity.

*Bijeljic et al*.^25^ and *Bijeljic et al*.^26^ solved the steady-state flow equations in three-dimensional images of the pore space of porous geo-materials. Solute transport was then numerically simulated by combining sub-pore scale advective motion along the streamlines determined earlier and molecular diffusion via a discretized space domain random walk. This method has been previously demonstrated to correctly reproduce Taylor dispersion in long cylindrical tubes and includes the same interactions among the geometric and hydrodynamic variables as in our study, albeit with a much more complicated geometry. The results tend to be in qualitative agreement with ours although differences in focus makes a full comparison difficult. For example, *Bijeljic et al*.^25^ reported that the high-τ branches of the observed transit-time distributions had an approximate power law form and showed a dependence of the exponent *f* on heterogeneity (in their Fig. 1) consistent with our results (Fig. 9). But the other branch of the transit-time distribution was not discussed, an omission due to their following the notion that only trapped and/or slowly moving solute particles contribute to non-Fickianity^11^. Our result that pore connectivity affects the 3^rd^ moment coefficient *F*_L_ but not the exponent *f*, suggests that fast moving particles may play a role too. The Eulerian velocity fields visualized in *Bijeljic et al*.’s^26^ Fig. 2 and the propagators of Figs. 5 and 6 suggest that velocities with a negative component in the nominal flow direction were present in these simulations. Propagator is a term referring to the probability distribution function of the displacements ζ experienced during a time δ*t* by solute particles uniformly distributed in the pore space. For infinitesimal δ*t*’s, the portion of the propagator corresponding to displacements greater than the diffusion displacement ζ_diff_ ≈ (2*D*_m_δ*t*)^1/2^ is essentially associated with the Eulerian velocities *u* = ζ/δ*t* (for ζ ≤ ζ_diff_, molecular diffusion may contribute significantly to the displacement). We estimate that *Bijeljic et al*.’s^26^ characteristic advective displacement < ζ_0_ > for δ*t* = 0.106 s (the shortest time interval considered) is on the order of 4.5 to 6.5 times ζ_diff_, thus implying that a portion of the negative displacements in the propagators of Bentheimer sandstone and Portland carbonate can be attributed to backward fluid velocities (see the first row of their Fig. 6bc). The bead-pack case is less conclusive (top of Fig. 6a) although *Cai et al*.^46^ observed negative velocities in similar bead-pack simulations. *Bijeljic et al*.’s^26^ observations confirm our result that the amount and magnitude of negative velocities increase with increasing material heterogeneity and decreasing pore connectivity (Portland carbonate is likely more heterogeneous and less well connected than Bentheimer sandstone). However, the effects of heterogeneity and connectivity are difficult to separate and quantify using the approach of *Bijeljic et al*.^26^.

## Conclusions

- We used a time domain random walk approach based on Poiseuille flow and Taylor dispersion to simulate passive solute transport in the backbone of heterogeneous and partially connected networks of cylindrical pores. We used the method of moments to extract the advection/dispersion characteristics of the simulated transport from the evolution with time of the plume of solute particles.
- Analysis of the first and second moments showed that the asymptotic regime was reached in all simulations and that the longitudinal dispersivity increased with increasing pore-size heterogeneity (*CV*) and decreasing pore connectivity (*Z* − *Z*_c_).
- One major finding was that the third moment was negative and that its magnitude grew linearly with time, unequivocally indicating that the asymptotic transport regime was intrinsically non-Fickian. Importantly, the non-Fickian behavior could not be attributed to diffusion traps (i.e., stagnant pores) since the simulations were restricted to the network backbones. Furthermore, we observed that the non-Fickian character was enhanced by increasing pore-size heterogeneity and/or reducing pore connectivity.
- The probability distributions of the Eulerian mean fluid velocities ${\hat{u}}_{i}$, the coefficients of Taylor dispersion ${\hat{D}}_{i}$ and the transit times ${\hat{\tau }}_{i}$ had complex non-Gaussian forms and were strongly affected by *CV* and *Z* − *Z*_c_.
- One important characteristic of the simulated Eulerian flow fields was the presence of negative velocities, the amount and magnitude of which increased with increasing *CV* and decreasing *Z* − *Z*_c_. As a consequence, backward and forward transit times had to be distinguished.
- The high-τ branch of the transit-time probability curves had a power law form, which was also observed in other studies and usually associated with non-Fickian behavior. The power law exponent decreased with increasing *CV* but was insensitive to changes in *Z* − *Z*_c_. On the other hand, pore connectivity did affect to the simulated non-Fickian behavior, which would not be possible if the high-τ branches were the sole contributors to non-Fickian dispersion^11,15,19,25,27^. We therefore conclude that the low-τ branches, often thought to exclusively embody Fickian dispersion, can in fact be partially responsible for non-Fickian transport.

### Data availability statement

The authors declare that the data are available.
